# Thermally treated zeolitic imidazolate framework-8 (ZIF-8) for visible light photocatalytic degradation of gaseous formaldehyde†

**DOI:** 10.1039/d0sc01397h

**Published:** 2020-05-21

**Authors:** Tianqi Wang, Yufei Wang, Mingzhe Sun, Aamir Hanif, Hao Wu, Qinfen Gu, Yong Sik Ok, Daniel C. W. Tsang, Jiyang Li, Jihong Yu, Jin Shang

**Affiliations:** School of Energy and Environment, City University of Hong Kong Tat Chee Avenue Kowloon Hong Kong China jinshang@cityu.edu.hk +852 3442 0688 +852 3442 7714; State Key Laboratory of Inorganic Synthesis and Preparative Chemistry, College of Chemistry, Jilin University Changchun 130012 China lijiyang@jlu.edu.cn jihong@jlu.edu.cn +86 431 8516 8608 +86 431 8516 8608; City University of Hong Kong Shenzhen Research Institute 8 Yuexing 1st Road, Shenzhen Hi-Tech Industrial Park, Nanshan District Shenzhen China; The Australian Synchrotron (ANSTO) 800 Blackburn Road Clayton VIC 3168 Australia; Korea Biochar Research Center, O-Jeong Eco-Resilience Institute (OJERI) & Division of Environmental Science and Ecological Engineering, Korea University Seoul 02841 Republic of Korea; Department of Civil and Environmental Engineering, The Hong Kong Polytechnic University Hung Hom Kowloon Hong Kong China dan.tsang@polyu.edu.hk +852 2334 6389 +852 2766 6045

## Abstract

The development of wide-spectrum responsive photocatalysts for efficient formaldehyde (HCHO) removal is highly desired yet remains a great challenge. Here we successfully converted zeolitic imidazolate framework-8 (ZIF-8), one of the most well-studied metal–organic frameworks (MOFs), from routine ultraviolet-driven to novel broad-spectrum-driven photocatalyst *via* a facile thermal treatment. The isocyanate groups (–N=C=O) formed in the thermally treated ZIF-8 (ZIF-8-T) is crucial in enabling the superior photocatalytic performance in formaldehyde degradation. Specifically, the best-performing ZIF-8-T sample showed around 2.1 and 9.4 times the HCHO adsorption amount and the solar photocatalytic degradation rate, respectively, of pristine ZIF-8. In addition, ZIF-8-T exhibited visible light (*λ* ≥ 400 nm) photocatalytic HCHO degradation performance, photo-converting 72% and nearly 100% of 20 ppm and 10 ppm HCHO within 1 hour, respectively. This work affords new insights and knowledge that inspire and inform the design and development of MOF-based photocatalysts with broad-spectrum responses for efficient air purification operations.

## Abstract 2

The isocyanate groups (–N=C=O) derived from –N=C– bonds in the organic ligands enables the thermally treated ZIF-8 to act as wide-spectrum photocatalyst.

## Graphical abstract

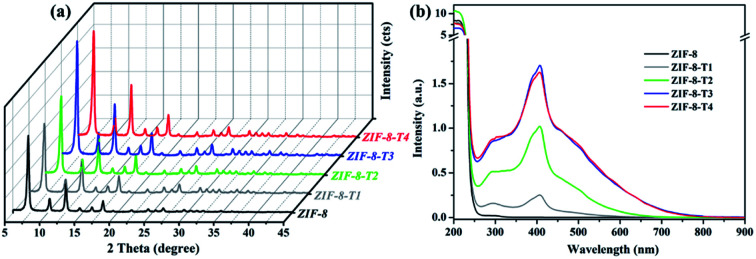

## Introduction

Formaldehyde (HCHO) is a critical indoor air pollutant that poses significant threats to human health.^1^ Driven by public concern, various technologies such as adsorption and photocatalysis have been developed for the removal of HCHO from indoor air.^2–5^ Adsorption is the most straightforward strategy for HCHO removal, but it, by itself, is unable to ultimately eliminate HCHO. On the other hand, photocatalysis can completely convert HCHO to harmless carbon dioxide and water under ambient conditions upon appropriate light irradiation. However, the efficiency of photocatalytic HCHO degradation is often not sufficiently high due to the limited HCHO adsorption on conventional photocatalysts such as TiO_2_ and g-C_3_N_4_,^6,7^ which have limited surface to bind pollutants. It is well acknowledged that adsorption is crucial for efficient photocatalysis because many photocatalytic reactions necessitate the short-range contact between catalysts and reactants. A porous structure in photocatalysts can not only improve HCHO adsorption, but also elevate photocatalytic efficiency due to the more abundant active chemical sites and the shorter electron-transfer distance compared with non-porous photocatalysts.^8–10^ A hierarchical porous structure is more attractive owing to the facilitation of mass diffusion and the improved light transmittance.^11–13^ Therefore, a porous photocatalyst is desirable for HCHO elimination.

One family of such promising candidate materials are metal–organic frameworks (MOFs). MOFs are a class of porous materials composed of metal ions and organic bridging ligands, which exhibit unique properties such as rich porosity, large specific surface area, abundant active centers, remarkable reusability, and tunable photocatalytic activity.^14–17^ One of the most outstanding advantages of MOFs over traditional semiconductors is the high designability at molecular level, which is enabled by rationally tuning metal ions or organic ligands. Therefore, MOFs have been successfully employed as a new class of photocatalysts towards various photocatalytic applications. For example, zeolitic imidazolate framework-8 (ZIF-8), one of the most well-studied MOFs, has been utilized for photocatalytic bacterial disinfection under simulated solar irradiation^18^ and degradation of methylene blue dye under UV light irritation.^19^ Hierarchical porous ZIF-8 has been readily prepared,^20,21^ showing a potential in facilitating pollutant elimination. However, the photocatalytic HCHO degradation by ZIF-8 has been rarely studied. Due to the wide bandgap energy,^18^ ZIF-8 can only be excited by UV light, which accounts for as low as 4% of solar spectrum.^22^ To better utilize natural sunlight and thus enable an efficient photocatalysis, a photocatalyst is highly desirable that can be responsive to visible light, which constitutes about 45% of sunlight.^23^

Doping (or co-doping) and dye sensitization are the common strategies to realize the visible-light activation of wide-bandgap photocatalysts.^24–26^ However, these strategies face limitations such as high material cost (*e.g.* upon transition metals used as dopants), high energy consumption (*e.g.*, doping temperature 700–1200 °C), and risk of secondary pollution (*e.g.*, metal/dye leakage). Recently, the important role of ligand types in extending the optical absorption of MOFs was discovered. For instance, with aminoterephthalate instead of terephthalate as linker, the UiO-66 MOF showed an increase in absorption band from 300 to 440 nm.^27^ More recently, Fu *et al.* demonstrated a NH_2_-MIL-125(Ti) MOF exhibited visible-light response by changing the ligand from terephthalic acid to 2-aminoterephthalic acid.^28^ These studies also emphasized the significant role of amine groups (NH_2_) within ligands in realizing visible-light photocatalysis on MOFs. On the other hand, isocyanate (–N=C=O) groups showed great potential to convert the UV-responsive semiconductors (*e.g.*, TiO_2_) into visible-light photocatalysts. For example, –N=C=O groups (2,4-diisocyanate, TDI) anchored on TiO_2_*via* –NHCOOTi– bonds could be excited by visible light and transferred electrons to the conduction band of TiO_2_, therefore, the optical absorption of TiO_2_/TDI complex was extended to visible light region, realizing photocatalytic dye degradation under visible light irradiation.^24,29^ Chen *et al.* also reported a –N=C=O groups-modified TiO_2_, which showed visible-light absorption and enhanced photocatalytic performance towards 2,4-dichlorophenol degradation.^30^ They explained it by a direct surface electron transfer from the lone pair electrons of N atoms and O atoms to the conduction band of TiO_2_. Fan *et al.* reported a silver isocyanate (AgNCO) photocatalyst synthesized by the precipitation of molecular isocyanate N=C=O^−^ and Ag^+^.^31^ The AgNCO photocatalyst exhibited visible-light photocatalytic degradation ability and superior stability. Upon visible light irradiation, the electrons transferred from Ag and pi bond to isocyanate group and converted it from N^2−^–C≡O state to N^−^=C=O state. Subsequently, electron-induced reactive species were generated for photocatalytic degradation. These exciting works motivate us to functionalize ZIF-8, a typical wide-bandgap and one of the most well-studied MOFs, to be a visible-light-driven photocatalyst by tuning its 2-methylimidazole (C_4_H_6_N_2_) ligand. We hypothesized that the –N=C– bonds containing 2-methylimidazole can be modified to generate the –N=C=O functional groups through oxidation. Therefore, we initiated to develop a facile strategy to enable ZIF-8 to exhibit visible light activity.

**Scheme 1:**
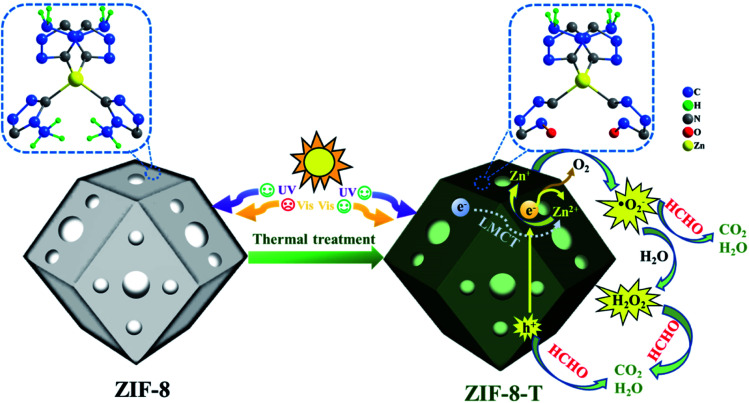
Schematic representation of ZIF-8-T for photocatalytic HCHO degradation.

Herein, for the first time, we functionalized hierarchical porous ZIF-8 nanocrystals as a visible-light-driven photocatalyst *via* a facile thermal treatment in air at a temperature as low as 200 °C (Scheme 1). The formation of new –N=C=O functional groups (derived from –N=C– bonds in the organic ligands *via* oxidation) was discovered in the thermally treated ZIF-8 (named ZIF-8-T), leading to an increase in the specific surface area and a substantially expanded light absorption range from UV to infrared. By coupling adsorption and photocatalytic oxidation, efficient HCHO elimination was achieved by ZIF-8-T under visible light and simulated sunlight. Considering the availability of various organic linkers and the possibility of modulating the composition of MOFs, we believe this study can open up new opportunities to develop efficient and wide-spectrum responsive MOF photocatalysts.

## Results and discussion

### Characterization of the as-made materials

**Fig. 1:**
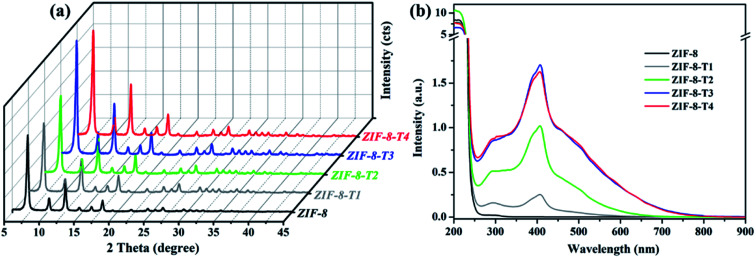
(a) PXRD patterns and (b) UV-vis DRS spectra of the as-prepared samples.

As shown in Fig. 1a, the powder X-ray diffraction (PXRD) pattern of the as-prepared ZIF-8 is in good agreement with that of previously reported ZIF-8,^32,33^ suggesting the successful synthesis of pure phase ZIF-8. The PXRD patterns of ZIF-8-T samples are consistent with that of pristine ZIF-8, indicating that the ZIF-8 framework was well preserved after thermal treatment. Notably, the PXRD peaks of ZIF-8-T products (especially ZIF-8-T3) are significantly stronger than that of ZIF-8, suggesting an improved crystallinity caused by the thermal treatment. No significant changes in the PXRD pattern of ZIF-8-T4 were observed when further prolonging the thermal treatment to 7 h. The lattice parameters of ZIF-8 before and after thermal treatment were estimated by analyzing the PXRD patterns, with *a* = 17.0130 Å for ZIF-8 and *a* = 17.0206 Å for ZIF-8-T-3. Therefore, the lattice structures of ZIF-8 remained almost unchanged upon thermal treatment. As shown in the ultraviolet-visible diffuse reflectance spectroscopy (UV-vis DRS) spectra (Fig. 1b), all ZIF-8(-T) samples exhibit strong absorption peaks at around 207 nm, which is attributed to the intra-ligand charge transfer, as suggested by the very similar absorption profile of 2-methylimidazole (H-MeIM) ligand (ESI Fig. S1†). Pure ZIF-8 also shows a broad absorption located at *ca.* 300 nm, which corresponds to the ligand-to-metal charge transfer (LMCT).^18^ After the thermal treatment, ZIF-8-T samples exhibit a remarkably improved light absorption especially in visible light region – a significant absorption at 420 nm together with the minor LMCT signal at 300 nm. In particular, the absorption range of ZIF-8-T3 photocatalyst even extends to above 700 nm, indicating a broad-spectrum-responsive characteristic. Compared with the recently reported MOFs-based visible-light photocatalysts (ESI Table S5†), which showed limited absorption in visible light region, the ZIF-8-T photocatalysts prepared in this work exhibited sufficient visible light absorption, with the absorption band being 715 nm. Thus, ZIF-8-T photocatalysts show great potential in using sunlight.

**Fig. 2:**
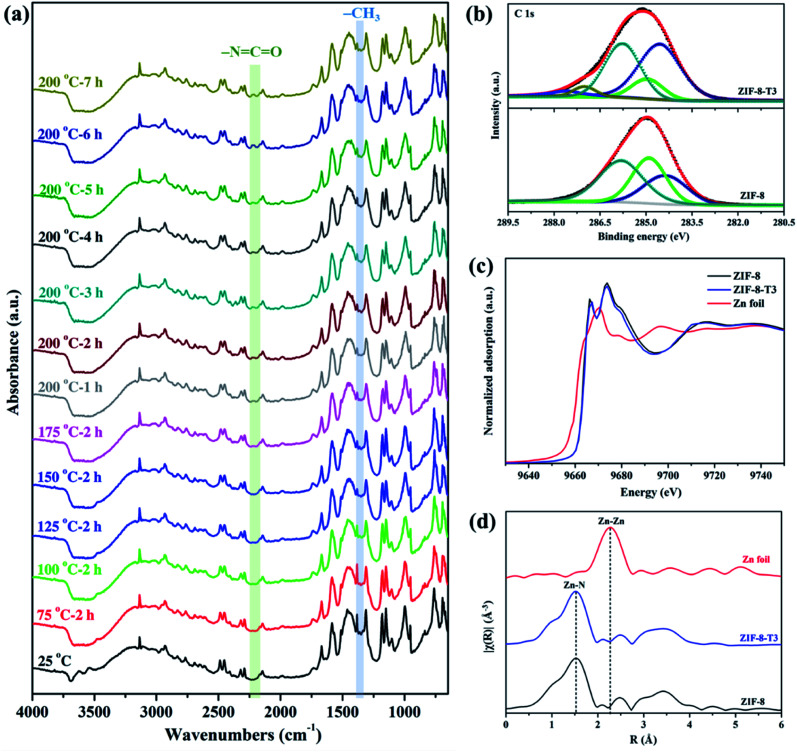
(a) *In situ* FTIR spectra of ZIF-8 recorded at different temperatures in air, (b) C 1s XPS spectra, (c) XANES spectra, and (d) Zn K-edge EXAFS spectra of ZIF-8 and ZIF-8-T3.

To evaluate the chemical structure changes of ZIF-8 during the thermal treatment, *in situ* Fourier transform infrared (FTIR) analysis was carried out. As demonstrated in Fig. 2a, the peak of methyl groups (–CH_3_) at 1380 cm^−1^ becomes weaker at 125 °C, and almost disappears when the temperature reaches 150 °C, indicating the dissociation of –CH_3_ groups. James and Lin also reported the disappearance of –CH_3_ groups when thermally treating ZIF-8 at a 300 °C in air or nitrogen.^34^ Meanwhile, an additional peak corresponding to isocyanate groups (–N=C=O) is observed at 2223 cm^−1^ in the FTIR spectra of the thermally treated ZIF-8 samples at 175 °C, and it becomes more remarkable at 200 °C with increasing thermal treatment time. The FTIR peak location of –N=C=O observed in this work matches well with the previous studies. For example, the peak of –N=C=O groups was detected at 2225 cm^−1^ in the FTIR spectrum of Cu/Zr-HMS catalyst at 200 °C during gas adsorption.^35^ The FTIR peaks of –N=C=O groups in the absorbed isocyanate compound on Cu-ZMS-5 were observed at 2240 cm^−1^ and 2204 cm^−1^ at 100 °C and 200 °C, respectively.^36^ The –N=C=O groups on the surface of TiO_2_ showed a FTIR peak at 2260 cm^−1^.^37^ The above results suggest ZIF-8 was gradually oxidized and the new –N=C=O groups were generated during the thermal treatment process in air. On the other hand, when ZIF-8 was treated under N_2_, its FTIR spectrum (ESI Fig. S2†) shows the absence of –N=C=O peak as well as the decrease in the intensity of –CH_3_ peak, revealing that only the dissociation of –CH_3_ groups occurs during the thermal treatment of ZIF-8 in inert atmosphere.

X-ray absorption spectroscopy (XPS) spectra of the as-prepared samples were recorded to elucidate the elemental compositions and valence states. As shown in Fig. 2b, the C 1s XPS spectrum of ZIF-8 can be divided into three peaks located at 284.4, 284.9, and 285.8 eV, which are attributed to C=C, C-sp^3^, and C–N bindings, respectively.^16^ As for the C 1s XPS spectrum of ZIF-8-T3, two additional peaks denoting C=O bonds and C=N bonds from –N=C=O groups are observed at 287.1 eV and 287.6 eV, respectively.^38^ The peak area of C-sp^3^ (284.9 eV) in ZIF-8-T3 becomes significantly smaller than that in the XPS spectrum of ZIF-8, suggesting the loss of –CH_3_ groups in ZIF-8-T3. In addition, the same valence state of Zn^2+^ in both ZIF-8 and ZIF-8-T3 was confirmed by the Zn 2p XPS peaks at 1021.6 eV (Zn 2p_3/2_) and 1044.8 eV (Zn 2p_1/2_), as shown in ESI Fig. S3.†

To understand the local geometric structure of ZIF-8 before and after the thermal treatment, X-ray absorption spectroscopy (XAS), a powerful technique to determine the coordination environment and valence state of the target atoms, was employed. The Zn K edge X-ray absorption near edge structure (XANES) of ZIF-8-T3 is almost overlapped with that of pristine ZIF-8 (Fig. 2c), suggesting the unchanged Zn chemical state after thermal treatment. As shown in the extended X-ray absorption fine structure (EXAFS) spectra (Fig. 2d), the presence of Zn–N bonds and the absence of Zn–Zn bonds in both ZIF-8 and ZIF-8-T3 samples are observed, indicating that Zn atoms are atomically dispersed and bonded with N atoms. As listed in the fitting results (ESI Table S1†), ZIF-8 and ZIF-8-T3 have very similar Zn–N coordination number (3.97 for ZIF-8 and 3.95 for ZIF-8-T3) and bond length (1.997 Å for ZIF-8 and 1.998 Å for ZIF-8-T3), suggesting that no changes occur to Zn–N bond. The energy dispersive spectroscopy (EDX) measurements (ESI Fig. S4 and Table S2†) show that the atomic content of C in ZIF-8 decreases from 68.2% to 56.2% with the heating time increasing from 0 to 7 h, while O content increases from nearly zero to 6.27%. The EDX results further reveal the loss of methyl group and the incorporation of oxygen into ZIF-8 framework after the thermal treatment. Considering the cleavage of C–N connection is more easily to take place due to its lower bond dissociation enthalpy (449 kJ mol^−1^) than that of C=C (602 kJ mol^−1^) and C=N (615 kJ mol^−1^),^39,40^ it is deduced that upon heating, the dissociation of –CH_3_ group from the imidazolate ligand and the breakage of C–N bond occur, together with the generation of –N=C=O bonds. On the basis of the above discussion, the mechanism of partial structure changes of the pyrolyzed ZIF-8 is proposed and shown in Scheme 1.

**Fig. 3:**
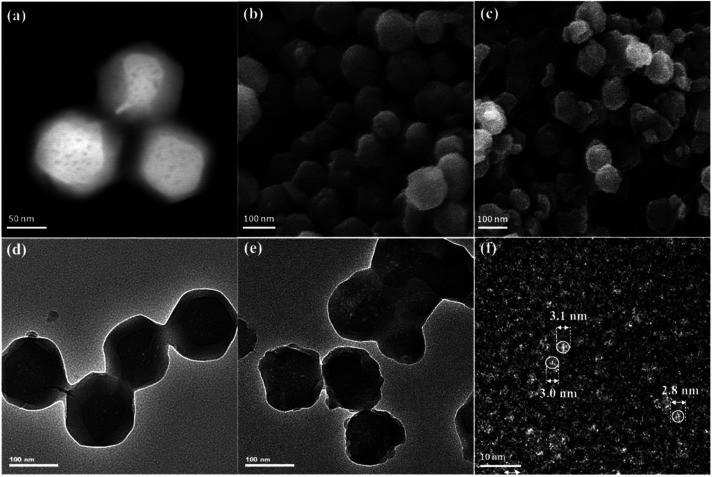
(a) Dark field TEM, (b) SEM, and (d) light field TEM of ZIF-8; (c) SEM, (e) light field TEM, and (f) high resolution TEM of ZIF-8-T3.

The morphologies of ZIF-8 and ZIF-8-T samples were investigated by scanning electron microscope (SEM) and transmission electron microscopy (TEM). As displayed in Fig. 3a and b, ZIF-8 nanostructures show a uniform rhombic dodecahedron morphology, with the particle size of approximately 90 nm, while the shape of ZIF-8-T3 particles is slightly irregular (Fig. 3d–f). Abundant mesopores are observed in both ZIF-8 and ZIF-8-T3 samples (Fig. 3a, d, and e), with the size being ∼3 nm in diameter, as shown in Fig. 3f. The presence of micro–mesopores in ZIF-8(-T) photocatalysts was favorably generated *via* the incomplete crystallization thanks to the relatively short reaction time (1 h) during the material synthesis, making a hierarchical porous structure supposed to facilitate guest adsorption and light transmittance.

**Fig. 4:**
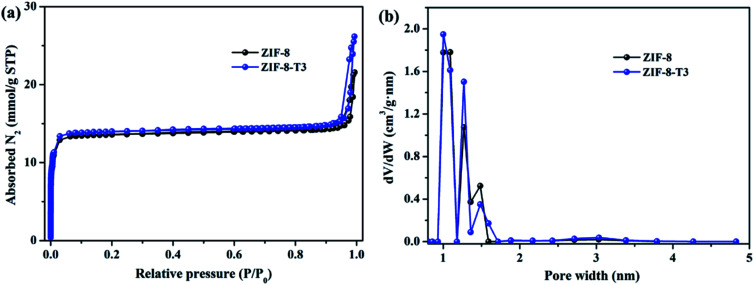
(a) N_2_ adsorption and desorption isotherms and (b) pore size distributions of ZIF-8 and ZIF-8-T3.

As mentioned above, the adsorption capacity of photocatalyst is important for the photocatalytic performance. Therefore, the porosity of ZIF-8 samples was studied by N_2_ adsorption–desorption isotherms conducted at 77 K (Fig. 4). As anticipated, the presence of hierarchical micro–mesopores is confirmed in all samples, as suggested by the sharp slopes of the isotherms appearing at low relative pressure range and the hysteresis loops at high relative pressure (*i.e.*, 0.92–1.0), respectively. The pore size distribution (Fig. 4b) also confirms the presence of micropores (1.0–1.7 nm) and mesopores (∼3.1 nm). It is well acknowledged that the hierarchical porous structure is favorable for gas diffusion and thus accelerates the subsequent gas adsorption and reaction. As shown in ESI Table S3,† the specific surface area of ZIF-8-T3 (1327 m^2^ g^−1^) is slightly higher than that of ZIF-8 (1212 m^2^ g^−1^), which is probably due to the formation of interrupted cavities in ZIF-8 framework caused by the broken bonds upon thermal treatment. The higher specific surface of ZIF-8-T can favor HCHO adsorption and offer more active sites for HCHO degradation.

**Fig. 5:**
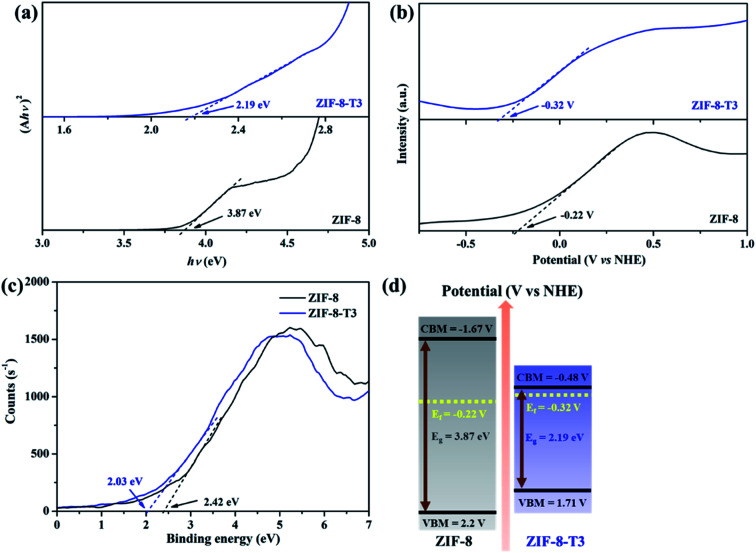
(a) Tauc plots, (b) Mott–Schottky plots, (c) valence band XPS spectra, and (d) band structures of ZIF-8 and ZIF-8-T3.

To characterize the band structures of ZIF-8 and ZIF-8-T3, Tauc plots, Mott–Schottky plots, and valence band XPS spectra were examined. As shown in the Tauc plots (Fig. 5a), the bandgap energy of ZIF-8 and ZIF-8-T3 is accordingly estimated to be 3.87 and 2.19 eV, respectively. The positive slopes of Mott–Schottky plots confirm the n-type semiconductor nature of ZIF-8 and ZIF-8-T3 (Fig. 5b), and thus their Fermi levels are close to the flat potentials, which are examined to be −0.22 V and −0.32 V (*vs.* NHE), respectively. As displayed in the valence band XPS spectra (Fig. 5c), the valence band (VB) maximum positions of ZIF-8 and ZIF-8-T3 are 2.63 eV and 2.61 eV below the Fermi level, respectively. The conduction band (CB) minimum positions are accordingly calculated to be −1.67 and −0.48 V (*vs.* NHE).^41^ Consequently, a diagram of the band structures is established as shown in Fig. 5d. The significant shift of CB and the moderate shift of VB lead to the narrowed bandgap of ZIF-8-T3, making the photo-excitation wavelength of ZIF-8-T3 fall into visible light region.

### Photocatalytic degradation of gaseous HCHO

**Fig. 6:**
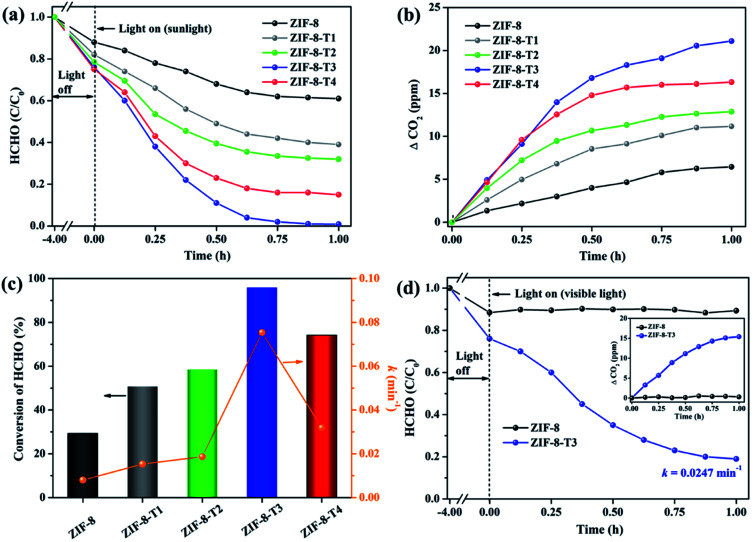
(a) Photocatalytic degradation of gaseous HCHO (20 ppm), (b) the corresponding CO_2_ evolution, and (c) HCHO conversion (evaluated by CO_2_ evolution in Fig. 6b) and kinetics over the as-prepared samples under simulated sunlight (AM 1.5); (d) photocatalytic degradation of gaseous HCHO (20 ppm) by ZIF-8 and ZIF-8-T3 under visible light (inset is the CO_2_ evolution curve).

In comparison with pure ZIF-8, ZIF-8-T catalysts exhibited substantially enhanced adsorption capacity and remarkably elevated photocatalytic degradation performance towards gaseous HCHO. Fig. 6a shows that about 11.6% and 24.0% of HCHO (20 ppm) were adsorbed by ZIF-8 and ZIF-8-T3, respectively, at equilibrium in dark condition. The HCHO adsorption capacity ZIF-8-T3 is calculated to be 1.51 mmol g^−1^, which is more than two times of that on pristine ZIF-8 (0.73 mmol g^−1^). The improved HCHO adsorption by ZIF-8-T3 is possibly attributed to the larger specific surface area and structure changes (*i.e.*, formation of –N=C=O bonds and dissociation of –CH_3_ groups). In addition, the less adsorption competition with CO_2_ may contribute to the enhanced HCHO adsorption by ZIF-8-T3, as suggested by its lower CO_2_ absorbed amount (0.45 mmol g^−1^ at 1 bar) than that of ZIF-8 (0.60 mmol g^−1^ at 1 bar) (ESI Fig. S5†).

The photocatalytic performance of the as-prepared samples was first examined under simulated sunlight (AM 1.5) irradiation. As shown in Fig. 6 and ESI Table S4,† only ∼29% of HCHO was photocatalytically degraded within 1 h by pristine ZIF-8. Impressively, a rapid decrease of HCHO concentration was observed once ZIF-8-T photocatalysts were employed. Among them, ZIF-8-T3 exhibited the highest photocatalytic HCHO degradation activity, with the pseudo-first-order kinetics (*k*) of 0.0754 min^−1^ and nearly 100% conversion of HCHO achieved within 1 h. The sunlight-driven HCHO degradation rate by ZIF-8-T3 is about 9.4 times of that by pristine ZIF-8. The efficient degradation of HCHO into CO_2_ and water by ZIF-8-T samples was corroborated by the evolution of CO_2_ (ΔCO_2_) (Fig. 6b), which was close to the theoretical ΔCO_2_ value corresponding to the degradation of HCHO. In addition, compared with ZIF-8-T3, although the microporous mZIF-8-T3 offers a larger specific surface area (ESI Fig. S6 and Table S3†), it shows a lower photocatalytic efficiency towards HCHO degradation (*k* = 0.0321 min^−1^) (Fig. S7†). This result suggests that the hierarchical micro–mesoporous structure of ZIF-8-T3 is beneficial to the photocatalytic performance, which can be ascribed to the better HCHO diffusion and light transmittance in ZIF-8-T3 photocatalyst.^12^ Zhang and co-workers have demonstrated that the presence of hierarchical pores facilitated the molecular reactants to reach the active sites on the porous interior.^13^ García–Benjume *et al.* have reported that the hierarchical porous structure of anatase photocatalyst allowed better light penetration and improved the photocatalytic performance of dye degradation.^11^

To investigate the responsiveness to light spectrum, the best-performing ZIF-8-T3 was then irradiated by visible light for HCHO degradation. As shown in Fig. 6d, ZIF-8-T3 exhibited an efficient visible-light-driven photocatalytic degradation of HCHO, with ∼72% decomposition of 20 ppm HCHO within 1 h (*k* = 0.0247 min^−1^). In addition, the effect of initial concentration of HCHO on the photocatalytic efficiency of ZIF-8-T3 was investigated. As shown in Fig. S8,† the photocatalytic HCHO degradation efficiency increased with decreasing HCHO concentration. Especially, nearly 100% HCHO removal (*k* = 0.0453 min^−1^) was achieved by ZIF-8-T3 within 1 h with the initial HCHO concentration of 10 ppm. By contrast, pristine ZIF-8 showed no visible light photoactivity. The photocatalytic performance of ZIF-8-T3 is significantly better than those of recently reported photocatalysts for HCHO degradation under similar experimental conditions.^6,42–45^ The stability and reusability of ZIF-8-T3 were investigated by repeating the photocatalytic HCHO degradation with recycled ZIF-8-T3, as shown in Fig. S9.† After each run, the photocatalyst was collected and degassed at 393 K for 12 h under vacuum to completely remove the absorbed HCHO as well as products from photocatalytic reactions. Importantly, ZIF-8-T3 photocatalyst exhibited excellent stability and reusability, as suggested by the almost unchanged photocatalytic HCHO degradation efficiency after 5 cycles. Therefore, the ZIF-8-T photocatalysts prepared in this work show a great potential for the real-world application in HCHO decomposition and abatement.

### Mechanisms of the enhanced photocatalytic activity of ZIF-8-T and reaction pathways of photocatalytic degradation of HCHO

**Fig. 7:**
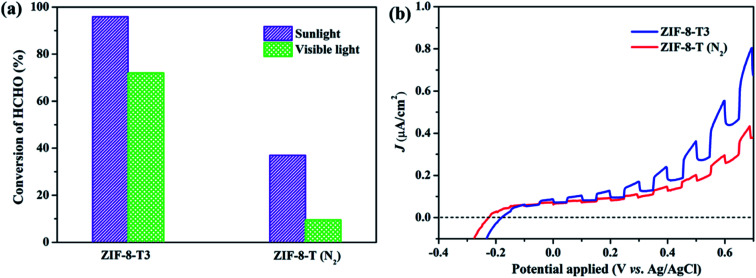
(a) Photocatalytic HCHO degradation efficiency by ZIF-8-T3 and ZIF-8-T (N_2_) under various irradiations and (b) voltammograms of ZIF-8-T3 and ZIF-8-T (N_2_) upon visible light irradiation.

As mentioned above, the major changes of the chemical structure of ZIF-8 after thermal treatment were the generation of –N=C=O functional groups and the dissociation of –CH_3_ groups. To understand which one is the dominant factor for the improved photocatalytic activity of the thermally treated ZIF-8, we then carried out the visible light photocatalytic HCHO degradation by ZIF-8-T (N_2_). ZIF-8-T (N_2_) was thermally treated under N_2_ atmosphere so that only the dissociation of –CH_3_ groups took place, whereas the formation of –N=C=O functional groups *via* oxidation was forbidden. As shown in Fig. 7a, in comparison with ZIF-8-T3 photocatalyst, ZIF-8-T (N_2_) sample exhibited much lower photocatalytic efficiency for HCHO degradation under both visible and simulated sunlight illuminations. Only 9.5% conversion of HCHO was achieved by ZIF-8-T (N_2_) under visible light. Although around 37% HCHO was degraded by ZIF-8-T (N_2_) under simulated sunlight, this efficiency is close to that of pristine ZIF-8 (∼29%). Moreover, the photocurrent intensity of the ZIF-8-T3 is significantly higher than that of the ZIF-8-T (N_2_) upon visible light irradiation at different voltages (Fig. 7b), revealing the better charge transfer ability of ZIF-8-T3. Therefore, it is the newly generated –N=C=O groups rather than the dissociation of –CH_3_ groups that dictates the outstanding photocatalytic capacity of ZIF-8-T photocatalyst, especially the visible-light-induced photocatalytic performance.

**Fig. 8:**
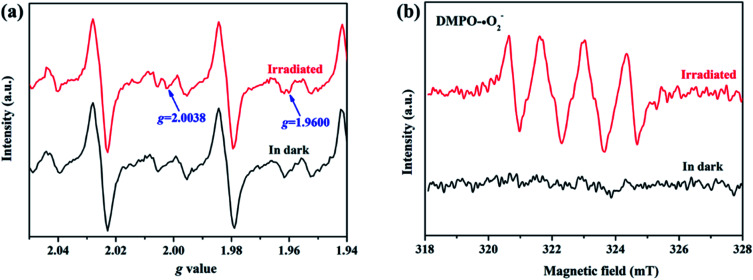
(a) EPR signals and (b) ˙O_2_^−^ DMPO spin-trapping EPR spectra of ZIF-8-T3 with and without simulated sunlight irradiation.

To elucidate the transfer pathway of photogenerated electron, we first monitored the valence state of Zn in ZIF-8-T3 upon light irradiation using electron paramagnetic resonance (EPR). The EPR spectra of ZIF-8-T3 recorded in air with and without sunlight irradiation are shown in Fig. 8a. In comparison with the unirradiated ZIF-8-T3, two new peaks appear at *g* = 2.0038 and *g* = 1.9600 in the EPR spectra of the irradiated one, which are ascribed to the absorbed ˙O_2_^−^ and the paramagnetic Zn^+^ centers, respectively.^46,47^ Therefore, it can be induced that under sunlight irradiation, the electrons generated by ligand (*via* LMCT) or semiconductor (from VB to CB) would transfer to Zn^2+^ to produce paramagnetic Zn^+^ sites, as shown in eqn (1)–(3). Then, ˙O_2_^−^ radicals are generated by electron transfer from Zn^+^ to O_2_ (eqn (4)). We also argue that the photogenerated electrons at semiconductor CB can directly react with O_2_ to form ˙O_2_^−^ radicals due to the more negative CB potential of ZIF-8-T3 (−0.48 V *vs.* NHE) than that of O_2_/˙O_2_^−^ (−0.33 V *vs.* NHE)^48^ (eqn (5)). As expected, four characteristic peaks of DMPO–˙O_2_^−^ adducts were clearly detected for ZIF-8-T3 under simulated sunlight irradiation, whereas no signal was observed for ZIF-8-T3 in dark (Fig. 8b). However, the generation of ˙OH radicals is thermodynamically forbidden due to the deficient VB potential (1.71 V *vs.* NHE) for the conversion of H_2_O to ˙OH (2.68 V *vs.* NHE). It is not surprising that negligible signals of ˙OH radicals were observed for ZIF-8-T3 with and without irradiation (ESI Fig. S10†).

**Fig. 9:**
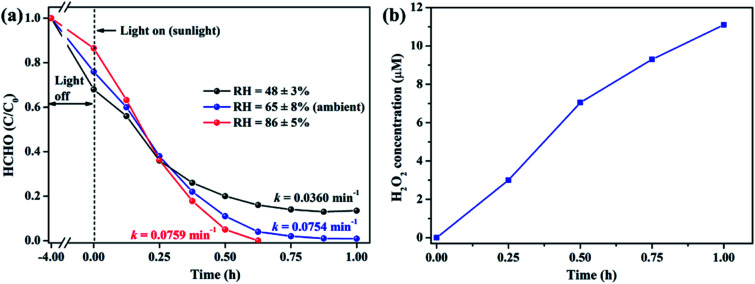
(a) Effect of relative humidity on photocatalytic degradation of gaseous HCHO (20 ppm) by ZIF-8-T3 under simulated sunlight and (b) concentration of H_2_O_2_ produced by ZIF-8-T3 in a liquid-phase system under simulated sunlight.

**Fig. 10:**
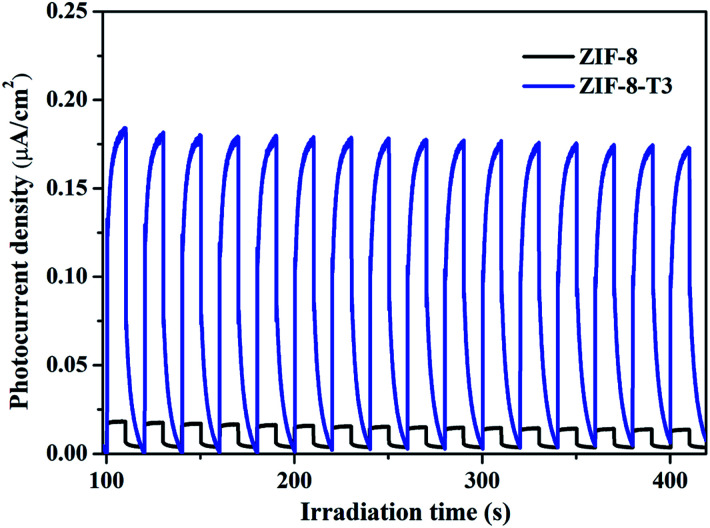
Photocurrent responses of ZIF-8 and ZIF-8-T3 under simulated sunlight.

It is well acknowledged that the performance of catalysts is strongly influenced by water species.^42^ To understand the effect of water species on the photocatalytic performance of ZIF-8-T3, the HCHO degradation experiments were carried out at low, ambient, and high relative humidity (RH). Fig. 9a demonstrates that the RH substantially affects the photocatalytic HCHO degradation efficiency. Although moisture competes with HCHO for adsorption by ZIF-8-T3, as revealed by the decreased HCHO adsorption amount at higher RH, the presence of moisture favors HCHO degradation significantly. This is ascribed to the important role of moisture in the generation of H_2_O_2_, which is active for HCHO degradation, as shown in eqn (7) and (8). The produced H_2_O_2_ by ZIF-8-T3 in a liquid-phase system was then detected by using *p*-hydroxyphenylacetic acid as the probe, with the total H_2_O_2_ concentration of 11.1 μmol L^−1^ irradiated by simulated sunlight for 1 h (Fig. 9b). It is noted that the photocatalytic HCHO degradation rate (*k* = 0.0754 min^−1^) at ambient RH (∼65%) is close to that (*k* = 0.0759 min^−1^) at higher RH (∼86%), suggesting ZIF-8-T3 photocatalyst can work efficiently at ambient condition. In addition, the VB potential of ZIF-8-T3 is positive enough to directly oxidize HCHO, as demonstrated by the study reported earlier.^7^ Therefore, ˙O_2_^−^, H_2_O_2_, and h^+^ are the major reactive species responsible for the photocatalytic HCHO degradation by ZIF-8-T3. Given the important role of electron-induced reactive species (*i.e.*, ˙O_2_^−^ and H_2_O_2_), the abundant photogenerated electron–hole charge pairs and the high electron transfer efficiency in ZIF-8-T3 (Fig. 10) are significantly favorable for the photocatalytic HCHO degradation performance. Based on the discussion above, the reaction pathways under sunlight irradiation are elaborated as the following equations and shown in Scheme 1:

1 ZIF-8-T3 + *hν* → e^−^ + h^+^

2 Ligand + Zn^2+^ → Zn^+^ (LMCT)

3 Zn^2+^ + e^−^ → Zn^+^

4 Zn^+^ + O_2_ → Zn^2+^ + ˙O_2_^−^

5 e^−^ + O_2_ → ˙O_2_^−^

6 ˙O_2_^−^ + HCHO → CO_2_ + H_2_O

7 ˙O_2_^−^ + H^+^ + H_2_O → H_2_O_2_ + O_2_ + OH^−^

8 2H_2_O_2_ + HCHO → 3H_2_O + CO_2_

9 h^+^ + HCHO → CO_2_ + H_2_O

## Conclusions

The wide-spectrum photocatalytic degradation of gaseous HCHO is for the first time achieved by the –N=C=O groups-functionalized ZIF-8 MOF, which is fabricated *via* a facile thermal treatment strategy at a relatively low temperature of 200 °C. The organic linkers in ZIF-8 are partially oxidized after thermal treatment, resulting in the generation of –N=C=O functional groups. The optical absorption band of ZIF-8-T is broadened from UV (∼325 nm) to visible (∼715 nm) region. Thanks to the introduction of –N=C=O groups into ZIF-8 backbones, efficient photocatalytic degradation of gaseous HCHO by ZIF-8-T is achieved upon sunlight and visible light irradiations. In addition, the hierarchical porous structure of ZIF-8-T facilitates the HCHO adsorption and light transmittance. Compared with pristine ZIF-8, ZIF-8-T photocatalyst shows more abundant charge carrier density and more efficient electron transfer, which are favorable for the photocatalytic performance. An LMCT process in ZIF-8-T during the photocatalytic reaction is established. The photogenerated reactive species including ˙O_2_^−^ radicals, H_2_O_2_, and h^+^ play the important role in HCHO degradation. This work provides valuable insights into the development of visible-light-induced MOFs photocatalysts and boosts the application of MOFs in air purification.

## Experimental

### Synthesis of hierarchical porous ZIF-8 and conventional ZIF-8

Hierarchical porous ZIF-8 was synthesized by following a reported procedure (no thermal treatment in this procedure)^49^ with minor modifications. Herein, we used 1/4 volume of reaction solutions (*i.e.* 50 mL) than the reported ones. In brief, 0.7332 g of Zn(NO_3_)_2_·6H_2_O was dissolved in 50 mL methanol as solution A, and 1.6225 g of 2-methylimidazole was dissolved in 50 mL methanol as solution B. Then, solution A was rapidly added into solution B within 30 seconds and stirred for 1 h. After centrifugation and washing with methanol for 5 times, the milky white hierarchical porous ZIF-8 product was obtained. Conventional microporous ZIF-8 was synthesized using the similar procedure as that of hierarchical porous ZIF-8, except that the mixed solution was stirred for 20 h.

### Synthesis of hierarchical porous ZIF-8-T and microporous ZIF-8-T

The hierarchical porous ZIF-8-T was obtained by thermally treating the as-prepared hierarchical porous ZIF-8 at 200 °C in air. To evaluate the effect of reaction time, a series of hierarchical porous ZIF-8-T samples were prepared by varying the thermal treatment time duration of 1, 3, 5, and 7 h. The products are denoted as ZIF-8-T1, ZIF-8-T2, ZIF-8-T3, and ZIF-8-T4, accordingly.

Since the pyrolysis atmosphere has significant influences on the chemical structure of products, we also thermally treated the hierarchical porous ZIF-8 under N_2_ atmosphere at 200 °C for 5 h. As anticipated, the oxidation of ZIF-8 and the generation of oxygen-related bonds were forbidden. The product is denoted as ZIF-8-T (N_2_).

To understand the role of hierarchical pores in ZIF-8-T on the adsorption and photocatalytic performance, we also fabricated microporous ZIF-8-T by using the as-prepared conventional ZIF-8 as precursor, with the same procedure as that of ZIF-8-T3. The product is named as mZIF-8-T3.

### Characterizations

Powder X-ray diffraction (PXRD) patterns were recorded using an X-ray diffractometer (X'Pert3 Powder, PANalytical, Netherlands) with Cu Kα radiation (40 kV, 40 mA). X-ray absorption spectroscopy (XAS) measurements were conducted at the Australian Synchrotron (ANSTO) to evaluate the chemical state and configuration of Zn. The morphologies of ZIF-8 photocatalysts were investigated by transmission electron microscopy (TEM) and high angle annular dark field scanning TEM (HAADF-STEM) conducted using a high-resolution transmission electron microscope (Tecnai F20, FEI Company, USA). Energy dispersive spectroscopy (EDX) was measured by an EDX detector (Oxford Aztec Energy X-MAX 50) equipped in a scanning electron microscope (FEI Quanta 450 FEG) to examine the chemical compositions of photocatalysts. The indium film was used for sample loading to eliminate the influence on carbon content from the conductive tape. The porosity of ZIF-8 samples was determined by a 3Flex Surface Characterization Analyzer (Micromeritics Instrument Corp., U.S.A.) using N_2_ at 77 K. Prior to the measurement, all samples were degassed at 423 K for 12 h under vacuum using a VacPrep Degasser (Micromeritics Instrument Corp., U.S.A.). To examine the chemical state of Zn during the photocatalytic reaction, electron paramagnetic resonance (EPR) signals of Zn were recorded with a Bruker E500 spectrometer at room temperature in air. Besides, EPR signals of spin-trapped paramagnetic species (˙O_2_^−^ and ˙OH) were measured with corresponding *N*,*N*-dimethyl pyrroline *N*-oxide (DMPO). *In situ* Fourier transform infrared (FTIR) spectra were collected with a FTIR spectrometer (NICOLET iS50, Thermo Scientific, USA). The light absorption profiles of photocatalysts were examined by ultraviolet-visible diffuse reflectance spectroscopy (UV-vis DRS) recorded with an Lambda 950 spectrometer (PerkinElmer, U.S.A.). Photoelectrochemical measurements including transient photocurrent responses, electrochemical impedance spectroscopy (EIS), and Mott–Schottky plots were carried out with a CHI 760E electrochemical workstation (Shanghai Chen Hua, China).

### Photocatalytic degradation of gaseous HCHO

Photocatalytic degradation of gaseous HCHO was performed in a 400 mL PLS-SXE300 Labsolar-IIIAG photoreactor (Perfectlight Co., Ltd, China). Typically, 50 mg of photocatalyst powder was uniformly dispersed on the watch glass inside the reactor. Then an appropriate volume of HCHO solution (37 wt%) was injected into the reactor to reach the initial HCHO concentration of *ca.* 20 ppm. Prior to illumination, the reactor was heated at 40 °C for 4 h to completely vaporize the HCHO solution and to reach the adsorption equilibrium. A 300 W Xenon lamp (Newport-67005, Oriel Instruments, U.S.A.) with appropriate filter(s) was vertically placed outside the reactor to provide visible light or simulated sunlight irradiation. The reaction temperature was maintained at 40 °C by a water circulation bath in all photocatalytic experiments. At given time intervals, the HCHO concentration was determined using a gas chromatograph (Shimadzu GC-2010, Japan) equipped with a flame ionization detector (FID). The kinetics of photocatalytic degradation of HCHO was determined by using the pseudo-first-order kinetic equation ln(*C*_0_/*C*) = *kt*, where *C*_0_ is the initial concentration of HCHO, *C* is the HCHO concentration at time *t*, and *k* is the apparent reaction rate constant.

## Conflicts of interest

There are no conflicts to declare.

## Supplementary Material

SC-011-D0SC01397H-s001
