# Supplementary material for: Thermally treated zeolitic imidazolate framework-8 (ZIF-8) for visible light photocatalytic degradation of gaseous formaldehyde
Source: Chem Sci. 2020 May 21;11(26):6670–81. doi: 10.1039/d0sc01397h (PMC8159372; doi:10.1039/d0sc01397h)
Supplement: SC-011-D0SC01397H-s001 [file SC-011-D0SC01397H-s001.pdf]

Electronic Supplementary Material (ESI) for Chemical Science.
This journal is © The Royal Society of Chemistry 2020

Supplementary Information

Thermally Treated Zeolitic Imidazolate Framework-8 (ZIF-8) for Visible Light Photocatalytic Degradation of Gaseous Formaldehyde

Tianqi Wang,‡[ac] Yufei Wang,‡[b] Mingzhe Sun,[ac] Aamir Hanif,[ac] Hao Wu,[a] Qinfen Gu,[d] Yong Sik Ok,[e] Daniel C.W. Tsang,[*f] Jiyang Li,[*b] Jihong Yu[*b] and Jin Shang[*ac]

[a]*School of Energy and Environment, City University of Hong Kong, Tat Chee Avenue, Kowloon, Hong Kong, China.*

[b]*State Key Laboratory of Inorganic Synthesis and Preparative Chemistry, College of Chemistry, Jilin University, Changchun 130012, China.*

[c]*City University of Hong Kong Shenzhen Research Institute, 8 Yuexing 1st Road, Shenzhen Hi−Tech Industrial Park, Nanshan District, Shenzhen, China.*

[d]*The Australian Synchrotron (ANSTO), 800 Blackburn Road, Clayton, VIC 3168, Australia.*

[e]*Korea Biochar Research Center, O-Jeong Eco-Resilience Institute (OJERI) & Division of Environmental Science and Ecological Engineering, Korea University, Seoul 02841, Republic of Korea.*

[f]*Department of Civil and Environmental Engineering, The Hong Kong Polytechnic University, Hung Hom, Kowloon, Hong Kong, China.*

‡ These authors contributed equally to this work.

Corresponding authors:

---

[*]Tel: +86 431 8516 8608, Fax: +86 431 8516 8608, Email: lijiyang@jlu.edu.cn (J.L.).

[*]Email: jihong@jlu.edu.cn (J.Y.).

*Tel: +852 2766 6045, Fax: +852 2334 6389, Email: dan.tsang@polyu.edu.hk (D.T.).

*Tel: +852 3442 7714, Fax: +852 3442 0688, Email: jinshang@cityu.edu.hk (J.S.).

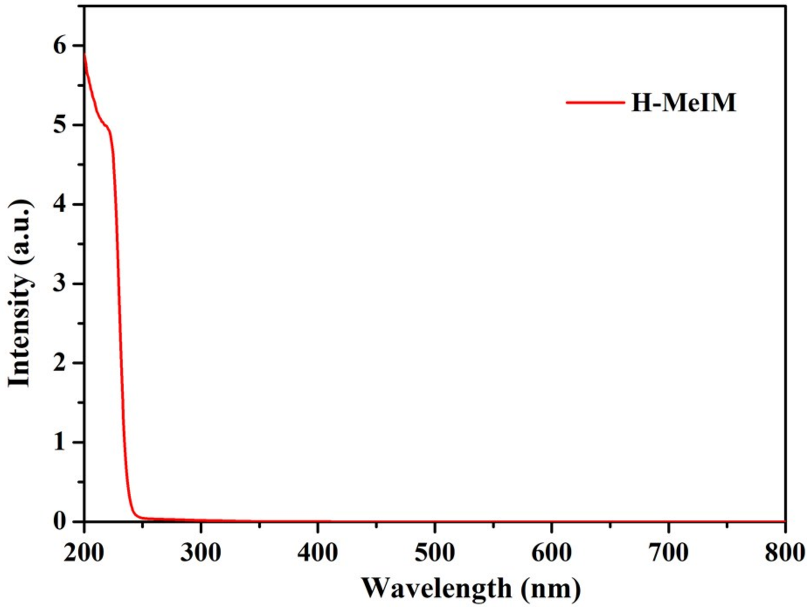

Fig. S1 UV-Vis DRS spectra of pristine H-MeIm.

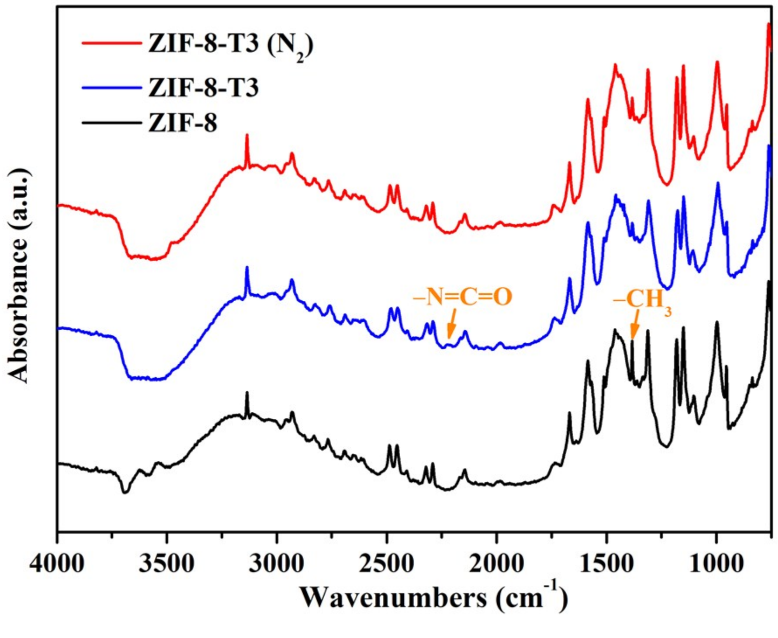

Fig. S2 FTIR spectra of ZIF-8, ZIF-8-T3, and ZIF-8-T (N_2).

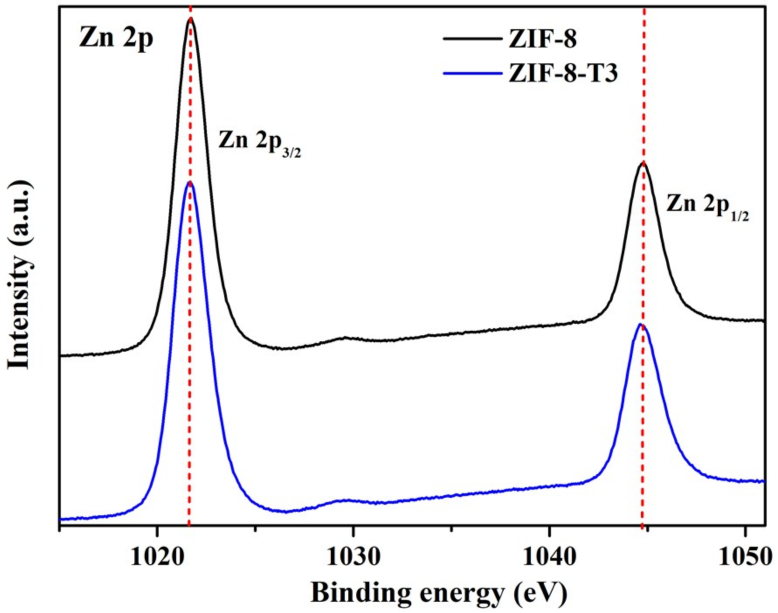

Fig. S3 Zn 2p XPS spectra of ZIF-8 and ZIF-8-T3.

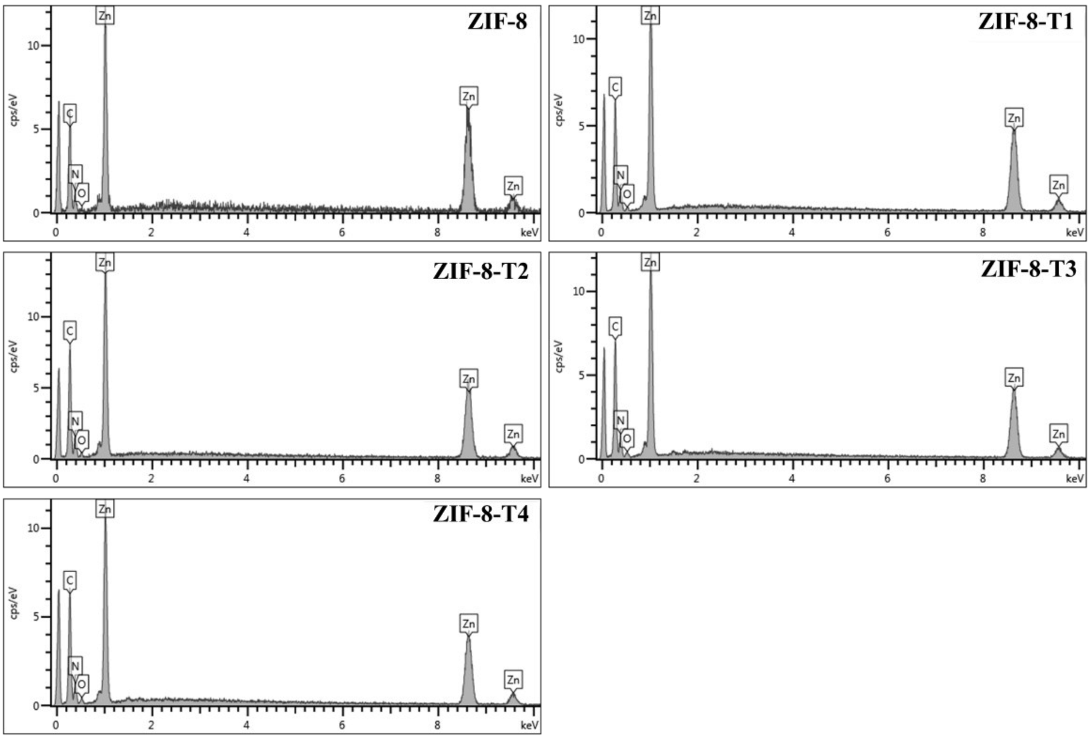

Fig. S4 EDX patterns of the as-prepared samples.

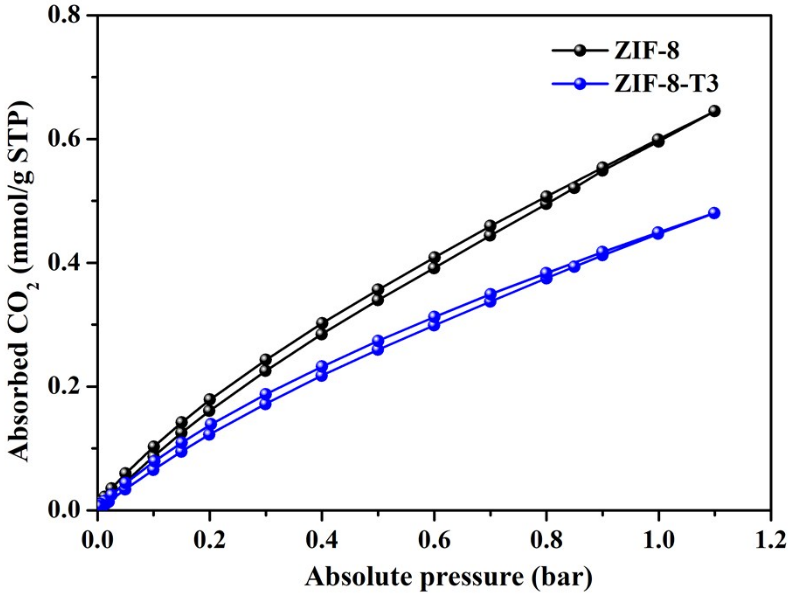

Fig. S5 (a) CO_2 adsorption and desorption isotherms of ZIF-8 and ZIF-8-T3.

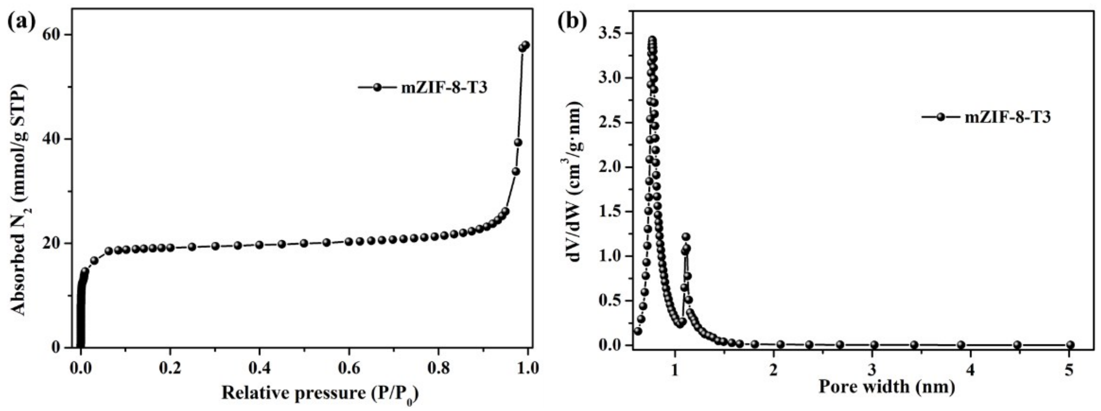

Fig. S6 (a) N_2 adsorption and desorption isotherms and (b) pore size distributions of micropore mZIF-8-T3.

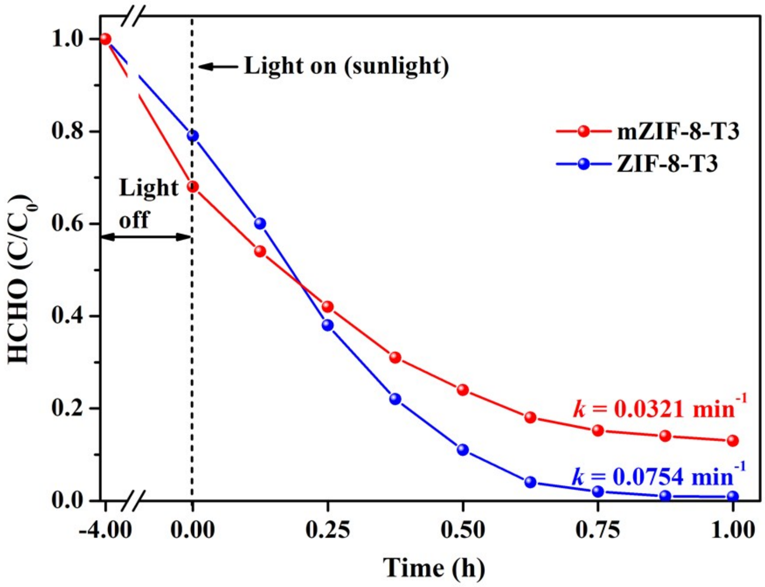

Fig. S7 Photocatalytic degradation of HCHO by mZIF-8-T3 and ZIF-8-T3 under simulated sunlight irradiation.

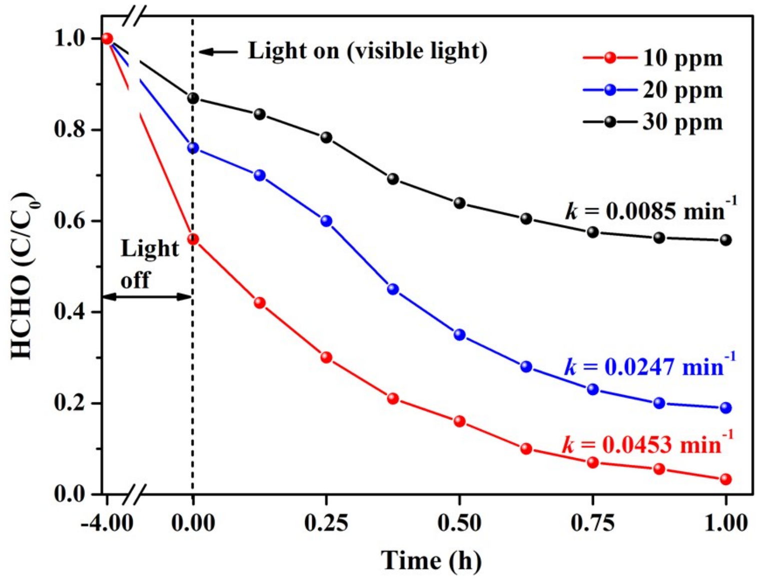

Fig. S8 Effect of HCHO initial concentration on the photocatalytic HCHO degradation by ZIF-8-T3.

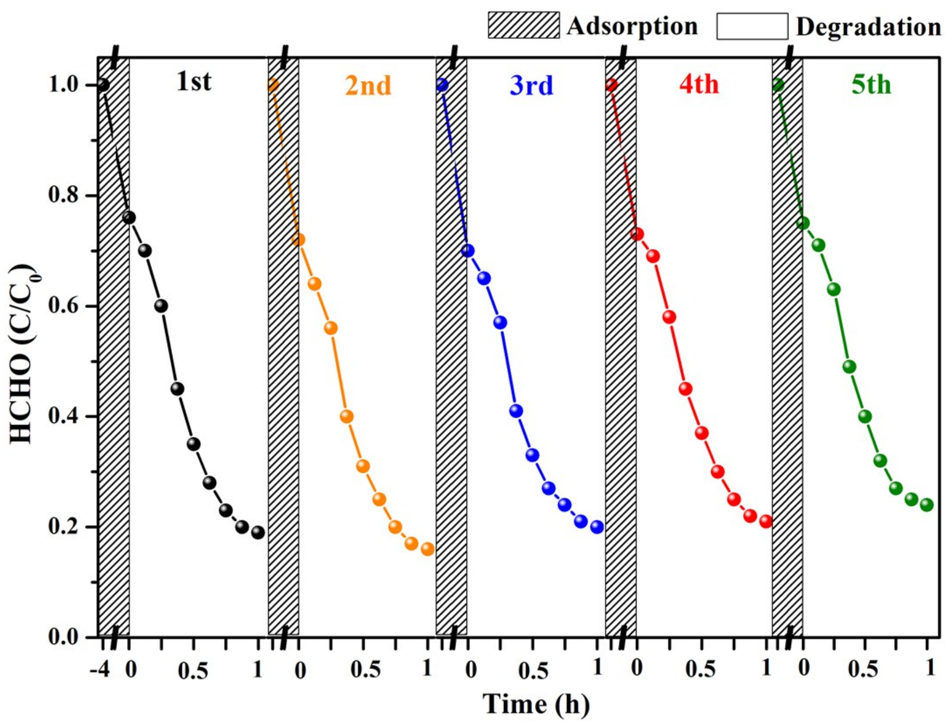

Fig. S9 Multiple-cycle runs of photocatalytic HCHO degradation by ZIF-8-T3 under visible light.

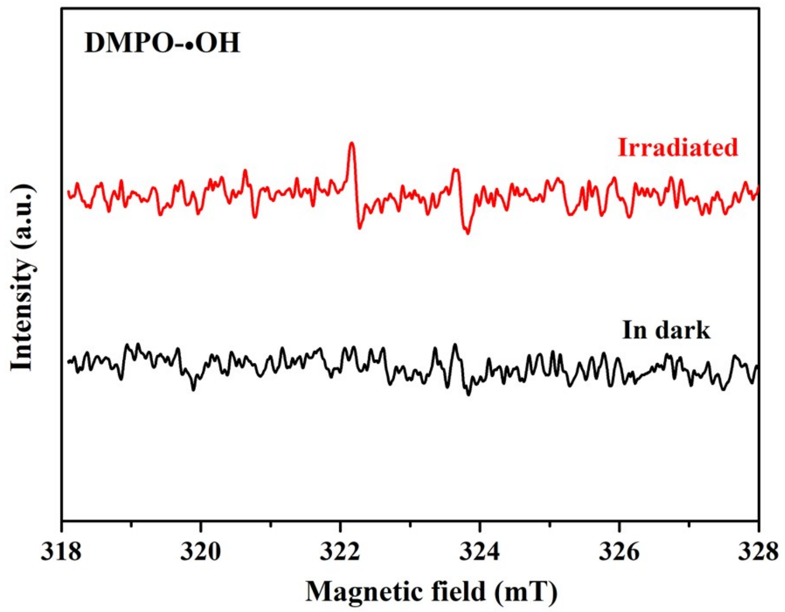

Fig. S10 •OH DMPO spin−trapping EPR spectra of ZIF-8-T3 with and without simulated sunlight irradiation.

Table S1 EXAFS data fitting results of Zn-N bond.

Sample	Coordination number	Bond length (Å)
**ZIF-8**	3.97	1.997
**ZIF-8-T3**	3.95	1.998

Table S2 Chemical composition (atomic %) of the as-prepared samples obtained from EDX measurement.

Sample	C K (Atomic %)	N K (Atomic %)	O K (Atomic %)	Zn K (Atomic %)
**ZIF-8**	61.86 ± 0.82	30.48 ± 0.40	0.42 ± 0.02	7.24 ± 0.16
**ZIF-8-T1**	59.69 ± 0.73	30.65 ± 0.45	2.25 ± 0.12	7.41 ± 0.21
**ZIF-8-T2**	56.30 ± 0.64	30.72 ± 0.45	5.62 ± 0.14	7.36 ± 0.18
**ZIF-8-T3**	56.17 ± 0.60	30.36 ± 0.31	6.26 ± 0.10	7.18 ± 0.12
**ZIF-8-T4**	56.19 ± 0.63	30.34 ± 0.39	6.27 ± 0.12	7.20 ± 0.16

Table S3 Porosity of the as-prepared samples.

Sample	Surface area S_{BET} ($m^2 \cdot g^{-1}$)	Total pore volume V_t ($cm^3 \cdot g^{-1}$)	Average pore size (nm)
**ZIF-8**	1212.20	0.67	1.61
**ZIF-8-T3**	1326.33	0.86	1.63
**mZIF-8-T3**	1669.86	2.00	1.05

Table S4 Photocatalytic HCHO conversion rate (evaluated by the CO_2 evolution amount) and kinetics over the as-prepared photocatalysts under simulated sunlight.

Sample	ZIF-8	ZIF-8-T1	ZIF-8-T2	ZIF-8-T3	ZIF-8-T4
**Degradation rate (%)**	29.31	50.74	58.50	95.90	74.21
**k (min^{-1})**	0.0080	0.0153	0.0187	0.0754	0.0319

Table S5 Comparison of the light absorption band of the reported visible-light photocatalysts and ZIF-8-T3.

Photocatalyst	Light absorption band (nm)	Ref.
g-C_3N_4	460	1
Fe_2O_3	575	2
Bi_2S_3	950	3
UiO-66-NH_2	480	4
MIL-125-NH_2	520	5
Pd@MIL-100(Fe)	440	6
NH_2-UiO-66(Zr/Ti)	460	7
CdS/MIL-101	560	8
NH_2-MIL-125(Ti)	525	9
ZIF-8-T3	715	This work

References

1. X. Wang, K. Maeda, A. Thomas, K. Takanabe, G. Xin, J. M. Carlsson, K. Domen and M. Antonietti, *Nat. Mater.*, 2009, **8**, 76-80.
2. D. H. Taffa, I. Hamm, C. Dunkel, I. Sinev, D. Bahnemann and M. Wark, *RSC Adv.*, 2015, **5**, 103512-103522.
3. H. Sun, Z. Jiang, D. Wu, L. Ye, T. Wang, B. Wang, T. An and P. K. Wong, *ChemSusChem*, 2019, **12**, 890-897.
4. T. W. Goh, C. Xiao, R. V. Maligal-Ganesh, X. Li and W. Huang, *Chem. Eng. Sci.*,

2015, **124**, 45-51.

5. S. Y. Han, D. L. Pan, H. Chen, X. B. Bu, Y. X. Gao, H. Gao, Y. Tian, G. S. Li, G. Wang and S. L. Cao, *Angew. Chem. Int. Edit.*, 2018, **57**, 9864-9869.
6. D. Wang and Z. Li, *Journal of catalysis*, 2016, **342**, 151-157.
7. D. Sun, W. Liu, M. Qiu, Y. Zhang and Z. Li, Chem. Commun., 2015, **51**, 2056-2059.
8. J. He, Z. Yan, J. Wang, J. Xie, L. Jiang, Y. Shi, F. Yuan, F. Yu and Y. Sun, Chem. Commun., 2013, **49**, 6761-6763.
9. H. Wang, X. Yuan, Y. Wu, G. Zeng, X. Chen, L. Leng, Z. Wu, L. Jiang and H. Li, J. Hazard. Mater., 2015, **286**, 187-194.
